# Economic burden associated with alcohol dependence in a German primary care sample: a bottom-up study

**DOI:** 10.1186/s12889-016-3578-8

**Published:** 2016-08-31

**Authors:** Jakob Manthey, Philippe Laramée, Steve Parrott, Jürgen Rehm

**Affiliations:** 1 Institute for Clinical Psychology and Psychotherapy, TU Dresden, Chemnitzer Str. 46, 01187 Dresden, Germany; 2 Institute for Mental Health Policy Research, Centre for Addiction and Mental Health (CAMH), 33 Russell Street, Toronto, ON M5S 2S1 Canada; 3 Department of Health Sciences, University of York, Heslington, York, UK; 4 Campbell Family Mental Health Research Institute, CAMH, 250 College Street, Toronto, ON M5T 1R8 Canada; 5 Institute of Medical Science (IMS), University of Toronto, Medical Sciences Building, 1 King’s College Circle, Room 2374, Toronto, ON M5S 1A8 Canada; 6 Department of Psychiatry, University of Toronto, 250 College Street, 8th Floor, Toronto, ON M5T 1R8 Canada; 7 Dalla Lana School of Public Health, University of Toronto, 155 College Street, 6th Floor, Toronto, ON M5T 3M7 Canada

**Keywords:** Cost of illness, Alcohol dependence, Primary care, Costs, Economic burden, Germany

## Abstract

**Background:**

A considerable economic burden has been repeatedly associated with alcohol dependence (AD) – mostly calculated using aggregate data and alcohol-attributable fractions (top-down approach). However, this approach is limited by a number of assumptions, which are hard to test. Thus, cost estimates should ideally be validated with studies using individual data to estimate the same costs (bottom-up approach). However, bottom-up studies on the economic burden associated with AD are lacking. Our study aimed to fill this gap using the bottom-up approach to examine costs for AD, and also stratified the results by the following subgroups: sex, age, diagnostic approach and severity of AD, as relevant variations could be expected by these factors.

**Methods:**

Sample: 1356 primary health care patients, representative for two German regions. AD was diagnosed by a standardized instrument and treating physicians. Individual costs were calculated by combining resource use and productivity data representing a period of six months prior to the time of interview, with unit costs derived from the literature or official statistics. The economic burden associated with AD was determined via excess costs by comparing utilization of various health care resources and impaired productivity between people with and without AD, controlling for relevant confounders. Additional analyses for several AD characteristics were performed.

**Results:**

Mean costs among alcohol dependent patients were 50 % higher compared to the remaining patients, resulting in 1836 € excess costs per alcohol dependent patient in 6 months. More than half of these excess costs incurred through increased productivity loss among alcohol dependent patients. Treatment for alcohol problems represents only 6 % of these costs. The economic burden associated with AD incurred mainly among males and among 30 to 49 year old patients. Both diagnostic approaches were significantly related to the economic burden, while costs increased with alcohol use disorder severity but not with other AD severity indicators.

**Conclusions:**

Our study confirms previous studies using top-down approaches to estimate the economic burden associated with AD. Further, we highlight the need for efforts aimed at preventing adverse outcomes for health and occupational situation associated with alcohol dependence based on factors associated with particularly high economic burden.

**Electronic supplementary material:**

The online version of this article (doi:10.1186/s12889-016-3578-8) contains supplementary material, which is available to authorized users.

## Background

Alcohol dependence (AD) is a prevalent disorder in high-income countries, with 3.4 % of the adult population in the European Union being alcohol dependent [1]. AD represents a major health burden for modern societies [2] because of high mortality rates [3–5], associated disability [6] and prevalent comorbidities [7–9]. The considerable health burden is also closely linked to an economic burden for the society. The economic burden is defined by the societal costs incurred by a given disease and is usually measured against a counterfactual scenario which assumes that the given disease does not exist [10]. The costs incurred by AD and heavy drinking were estimated to amount to about 1 % of the European gross domestic product [11]. For Germany, the economic burden related to AD has not been estimated yet, but the burden associated with alcohol in total were also found to be around 1 % of the national gross domestic product [12, 13].

While a number of other studies have estimated the economic burden of alcohol in the EU in general, and Germany in particular e.g. [12, 14–16], studies on the economic burden of AD are more sparse as indicated in a recent review [17]. The burden of AD was found to be related to direct health care spending, e.g. for hospitalizations and medications, as well as to indirect costs associated with unemployment and absenteeism. The authors call for more cost-of-illness studies as they identified several shortcomings in the data. More detailed estimates are necessary to justify public spending, e.g. for planning and implementing public health programs to reduce the burden of addiction.

Cost-of-illness studies usually lack empirical assessments of health care utilization and therefore combine aggregated data with alcohol-attributable fractions (top-down method) to determine the share of alcohol consumption or alcohol dependence in cause-specific mortality and hospitalizations [2]. However, this approach is limited by a number of assumptions. First, alcohol-attributable fractions denote the proportion of cases attributable to all alcohol consumption by definition, usually derived from the distribution of average drinking levels and associated relative risks [18]; thus, they cannot be used to evaluate costs related to certain drinking patterns or alcohol-related diagnoses without being adjusted to the relevant category e.g. [11]. Second, it is assumed that risk functions employed in the calculation of alcohol-attributable fractions, developed from published meta-analyses based on systematic literature reviews that included international literature, can be applied to specific countries. Third, aggregate data usually include a number of estimates of different sources and thus reproduce their measurement errors.

Cost-of-illness studies that collect individual data (bottom-up method) do not rely on these assumptions and have thus the advantage of directly calculating the economic burden, making them a benchmark against which other studies using the top-down method can be compared with. Additionally, unlike studies relying on aggregate data, studies employing the bottom-up method generally allow for a more detailed calculation and separation of costs for specific subgroups for which population-attributable fractions are not available. The economic burden of a given disease is estimated by comparing mean costs of diseased and healthy individuals, which results in excess costs. While this approach suits well to determine costs due to morbidity and disability, it can hardly assess costs related to mortality, which should be considered [10] as they make up a substantial share of the economic burden associated with AD [11].

To date, previous studies have used bottom-up approaches to estimate the economic burden for some diseases e.g. [19, 20]; however, for AD, studies using this method in sufficiently large and representative samples are very sparse [for a systematic review of international cost-of-illness studies, see [11], for reviews of European cost-of-illness studies, see [17, 21]. Thus, using a bottom-up approach, the present studies’ primary aim was to estimate the economic burden of AD and to compare those estimates with previous top-down estimations, which suggest that indirect costs make up at least half of AD related costs in most countries [2, 11].

Previous estimates also indicated that 70 % of the economic burden related to alcohol consumption in Germany were caused by males [12], which is very similar to the sex ratio in AD diagnoses [1]. However, as this proportion varied considerably across various cost categories, a sex-stratified examination of the economic burden was considered important. Similarly, age was regarded a relevant factor the economic burden because the course of AD peaks at early ages [22] and interactions of AD-associated somatic comorbidities with age have been suspected [23].

For the purpose of estimating the economic burden associated with AD, we analysed a sample of German primary care patients from the ‘Alcohol Dependence in Primary and Specialist Care in Europe’ (APC) study [7, 24]. We identified two important AD characteristics that could influence the economic burden associated with AD. First, previous reports of the APC study showed that patients diagnosed with AD either through a standardized instrument assessing DSM-IV (Diagnostic and Statistical Manual of Mental Disorders, fourth edition) diagnoses [25] or through clinical judgements from the treating general practitioner (GP) made up two relevant but largely distinct groups of alcoholics [7]. As GP diagnosed patients were older and reported more comorbidities than patients with DSM-IV diagnoses, separate cost examinations for both diagnostic groups seemed reasonable. Second, the AD associated economic burden was expected to vary by AD severity as measured by the number of DSM-5 (Diagnostic and Statistical Manual of Mental Disorders, fifth edition) alcohol use disorder (AUD) criteria [26], drinking levels [27], or treatment for alcohol problems [28] because these indicators were found to be related to adverse mental and/or physical consequences as well as disability and mortality [4, 23, 28–30]. It may seem counterintuitive to consider alcohol treatment as indicator for severity but low treatment rates [31] and increased degree of impairment [32] suggest that only the most severe cases seek treatment. Given the associations with comorbidity, disability and mortality, it was expected that health care spending and impaired productivity would increase with AD severity. Thus, additional analyses should serve to measure the impact of the diagnostic approach and of different indicators of AD severity on the economic burden associated with AD.

In conclusion, the present studies’ primary objective was to estimate the cost-of-illness of AD patients, from a societal German primary care perspective using a bottom-up approach. The results were compared to previous estimates of German cost-of-illness studies of alcohol that used a top-down approach. In addition, this study stratified the results by sex and age, and performed additional analyses to examine the impact of AD characteristics (diagnosis and severity).

## Methods

### APC study design and participants

The APC study was designed to gain knowledge about the distribution, recognition, and treatment of AD in European primary health care settings. Previous results of the study indicate that AD among primary care patients was characterized by low socioeconomic status, unemployment, co-morbidities, mental distress, and disability [32]. For the study, a representative sample of GPs was drawn in six European countries (Germany, Hungary, Italy, Latvia, Poland, and Spain), after approval was obtained from the concerning ethic committees in all countries (Germany: approval gained on August 28, 2012; reference number: EK 207072012).

GPs assessed patients aged 18–64 on one day or more than one consecutive days using a brief questionnaire, which focused on the patients’ health and included alcohol-related questions as well as present, and lifetime AD diagnoses. A probability sample of all assessed patients was then drawn while oversampling patients perceived to have alcohol problems or AD by their GP. Sampled patients were further interviewed using a range of standardized instruments including the Composite International Diagnostic Interview CIDI; [33] which is the gold standard in assessing DSM-IV diagnoses [34]. It was previously shown that both CIDI and GPs diagnosed a similar proportion of individuals, with differences in age and comorbidities and overall little overlap between both groups [7]. As both approaches were judged valid, they were combined into a mutual AD category. Respective diagnoses were based on the 12-month time frame prior to the interview. The CIDI also assessed the patients’ drinking behavior with a quantity-frequency approach [35], which could be combined to form daily drinking levels as measured in standard drinks per day (one standard drink equals 10 g pure ethanol).

Furthermore, the patient interview also included the World Health Organization Disability Assessment Schedule (WHODAS) 2.0 [36] to assess sociodemographic variables and the degree of disability, the Kessler Psychological Distress Scale (K10), and a service use questionnaire adapted from the United Kingdom Alcohol Treatment Trial (UKATT) [37], which can be found in Additional file 1.

The German population sample of the APC study was identified by primary health care GPs from the ‘National Association of Statutory Health Insurance Physicians’ (“Kassenärztliche Bundesvereinigung”) (response rate at GP level: 36.7 %) who were practicing in two German regions: Berlin-Brandenburg (46.1 % of all GPs) and eastern Saxony (53.9 % of all GPs). Interviews with patients were conducted mainly via telephone (response rate at patient level: 75.7 %) between March 2013 and January 2014. All patients gave informed consent prior to being interviewed. A more detailed description of the APC study, its sampling design and instruments used can be found elsewhere [7, 24].

### Cost assessment

In order to determine the costs for each patient, we combined individual resource use and productivity data with respective unit costs. For example, in order to determine the costs of GP visits, we multiplied the number of visits with the costs of a single visit. All resource use data was collected via the patient interview and included hospital attendances, general practice visits, home care by healthcare and support professionals, medication use, and alcohol services use. If respondents felt that their type of contact was not part of the provided categories, they could specify the number of ‘other’ contacts. Most of these responses could be classified into existing or new categories based on their specification. If the specification was missing, the contacts were not included in determining respective costs.

Additionally, productivity data was also assessed through the interview and encompassed questions on the patients’ employment status and their absenteeism in the workplace. Most of these data were assessed using the UKATT questionnaire [37]. All questions in this instrument referred to a six months period prior to the interview, which was the default period in the original version of the questionnaire. We considered this period sufficiently long to gather important information about health care utilization and productivity, while being short enough to keep potential memory bias at a minimum.

These resource use and productivity data were then combined with German unit costs following a bottom-up approach, in compliance with the guidelines of the ‘Working Group Methods in Health Economic Evaluation’, which lists standard unit costs for health economy evaluations in Germany [38]. The majority of unit costs could be taken from their publication, while the remaining unit costs were taken from statistics and publications by official agencies or health insurance companies. All costs were updated to 2014-€ using the consumer price index [39]. For health care unit costs, we used health care specific price changes; for costs related to productivity, overall price changes were used. Unit costs in 2015-€ were deflated using the average rate from the first 10 months of the year 2015. A detailed description of all resource use components, their updated unit costs, and related sources are presented in Additional file 2: Web Table 1.

#### Direct costs

##### Hospital attendances

For hospital attendance rates, we assessed up to three (limit given by UKATT questionnaire) department-specific inpatient admissions, outpatient visits, day case surgery attendances, and accident and emergency attendances. Unit costs were derived from the guidelines for health economic evaluation in Germany [38]. For inpatient and outpatient visits, we used department-specific unit costs and mean costs if department-specific unit costs could not be assigned to the reported department. In Germany, separate day case surgery attendances generally do not exist and were thus rarely reported by the patients (6 % with at least one attendance). Day case surgery and outpatient visits were valued alike. Unit costs for accident and emergency attendances were equal to mean inpatient admission costs for patients who were admitted to the hospital following the treatment. For patients who received treatment in the accident and emergency department only, the respective department-specific outpatient unit costs were used. Therefore, all hospital-related costs could be grouped as either inpatient or outpatient costs.

##### General practice visits

We assessed the number of surgery and home visits, as well as practice nurse contacts (at the surgery) for each patient, while GP and nurse contacts were collapsed into a single category. Unit costs for personal patient contacts and home visits were taken from the standard evaluation criteria (Einheitlicher Bewertungsmaßstab) [40] – a catalogue on the costs of services reimbursed by the statutory health insurance to the respective health professionals. As appropriate in the German context [38], these costs had to be adjusted to the share of private health insurances in outpatient health care settings. For this reason, we updated the ratio of the share of patients covered by statutory health insurance [41] to the share of total revenue made by statutory health insurance companies [42], which resulted in a factor of 1.03 – smaller than the factor used by Krauth and colleagues [38] (1.11).

##### Home care by healthcare and support professionals

In this section, we asked patients for the number of home contacts by health professionals, including occupational therapists, support workers, social workers, community psychiatric nurses, district nurses, and other professionals, where patients specified using the service of physiotherapists and alternative practitioners. Unit costs for these services were drawn from different sources: For services provided by occupational therapists, we referred to the mean cost of various services at the patients’ home remunerated by different statutory health insurances in Berlin [43], after being weighted by the share of patients in the respective insurance companies [44], and adjusted with the above specified ratio for the share of private health insurances. Costs of a home session with a physiotherapist are based on the guidelines for health economic evaluations in Germany [38], and with a home visit charge added [45]. Unit costs for home visits from alternative practitioners were taken from a separate remuneration system which provides commonly used charges for different treatments [46]. Contacts with other health care professionals (e.g. social workers) were valued using the mean costs of occupational therapists, alternative practitioners, and physiotherapists. Lastly, the costs of paid homemakers providing household support were determined using the mean gross income of other labour forces [47].

##### Medication use

For prescribed medication, we assessed brand names, size of each dose (in mg), frequency of doses per day, and period of intake of up to five medications per patient (limit was predetermined by the UKATT questionnaire and was reached by 7.5 % of all patients). The cost of each medication was determined by multiplying costs per day with period of intake. While period of intake was directly assessed, costs per day were based on costs per dose (estimated by identifying price and size of available medication packages in the red list [48] or online pharmacies) and the reported dose or, if missing, the defined daily dose [49]. Additional file 2: Web Figure 1 describes these steps in detail (see Additional file 2).

Taken together, two core variables were needed to calculate medication costs: a) medication costs per day multiplied with b) period of medication intake. This data was only available in association with 37 % of all reported medications, mainly because the exact brand names were not given for 61 % of all reported medications, but instead their prescribed purpose or another reference category (e.g. hypertension medication, contraceptive, etc.). Because assessment of costs per package without a valid trade name were not feasible, the respective costs per day were unavailable. Therefore, we imputed the costs per day by using the *median* of the respective costs of similar medications of the same category identified via the ‘Anatomical Therapeutic Chemical’ code. Using the median allowed us to impute conservatively and be less affected by outliers of very costly medications. A similar procedure was applied to missing values of period of intake (5.7 % of all reported medications), for which the *mean* period was imputed among the same category of drugs. One case reported using a very costly drug (calculated costs: 315659.30€ in the 6-month observed period). In order to make sure that this outlier would not overly inflate the medication costs, we decided to adjust the costs to 150 % of the second highest single medication cost (adjusted costs: 7328€).

##### Alcohol specific services use

Alcohol specific individual or group contacts with GPs or their nurses, with alcohol agencies, at residential rehabilitation institutions, at hospitals, with self-help groups, and other contacts were assessed. All reported contacts were grouped into GP contacts, inpatient admissions (in residential rehabilitation institutions or hospitals), outpatient visits (at hospitals or alcohol agencies), counselling (at alcohol agencies, e.g. with psychotherapists) and group therapies. Alcohol-related GP contacts were valued as any other GP visit with unit costs taken from the standard evaluation criteria [40], and adjusted for the share of private health insurances (as described above). The unit costs for inpatient and outpatient treatment were retrieved from the hospital based remuneration system for psychiatry and psychosomatic departments, referring to the daily mean remuneration for inpatient and outpatient treatment of AD, respectively [50]. Costs were determined based on the number of nights spent in the hospital or based on the number of semi-residential contacts, for inpatient and outpatients visits respectively. For alcohol counselling contacts, unit costs were based on contacts with psychotherapists and were taken from the German guidelines for health economic evaluations [38]. Unit costs of a single group therapy session were considered equal to the opportunity costs of one-hour leisure time of both the patient and the therapist. Opportunity costs were assessed via the gross average wage in Germany in 2014 [47].

#### Indirect costs - productivity losses

We considered absenteeism, unemployment, and disability or early retirement as indirect costs. First, respondents were classified as employed (paid work or self-employed), unemployed (but job seeking), or disabled/in early retirement (including unemployment due to health reasons) based on responses given in the WHODAS 2.0 sociodemographic section. Patients pursuing non-paid work, studying, or homemaking in their own home were not assigned any productivity-related costs, representing 17.2 % of all studied cases. The costs related to absenteeism among gainfully employed patients were estimated using the friction cost approach [51] and a frictional period of 49 days [38]. We referred to mean gross costs of one working hour multiplied with the mean working hours per day in 2013 [52] to assess the costs of one absent day. Costs related to unemployment could be retrieved from another study which took into account paid benefits, reduced tax revenue, and reduced insurance fees within statutory health and retirement insurances [53]. We manually estimated costs related to unemployment due to health problems, disability, or early retirement using the mean paid benefits for persons with reduced working capacity [54] in addition to costs of reduced tax and fee revenue per unemployed person according to the previously mentioned study [53]. This estimation assumes similar patterns of reduced spending among the disabled or early retirees compared to other unemployed patients. Although the classification of employment status only refers to the time of the interview, we generalized this classification to the six months prior to the interview in order to be in line with the other measures on direct and indirect costs. We did not include costs due to premature mortality because the design of this study did not allow for the assessment of this parameter.

In addition to calculating productivity-related costs, we also present descriptive statistics on presenteeism, i.e. reduced capacity while being at work. We did not include a standardized measure to directly determine the respective costs but collected information approximating this concept: number of days with reduced or cut-back work due to any health condition (WHODAS 2.0); number of days with at most half of the usual capacity due to mental distress (K10); number of days with affected productivity at work due to alcohol use (UKATT service use questionnaire). These measures are presented descriptively in order to grasp the magnitude of lost productivity while being at work.

### Statistical analyses

As contextual information, descriptive statistics for sociodemographic variables and the WHODAS sum score as indicator for disability were presented for patients with AD diagnoses and for those without. We tested for statistical differences by diagnostic status via negative binomial regression (for the WHODAS sum score) and via Chi^2^-Tests (for the remaining binary variables). The sampling design implied patients with alcohol problems or AD to be overrepresented in the interviewed sample. This distortion was accounted for by weighting all analyses with the inverse sampling probability.

#### Primary aim: estimation of economic burden of AD

The economic burden associated with AD was determined via excess costs, which were calculated as mean difference between patients with and without AD diagnosis by sex in the following sectors of direct and indirect costs: inpatient admission, outpatient visit, any GP visit, prescribed medication, home care, alcohol treatment, absenteeism, unemployment, disability/early retirement. Given the right skewed distribution of cost variables, significant differences were tested using age-adjusted negative binomial regressions for all count variables (most cost variables). Decisions for or against utilization of zero-inflated models for variables with excess zeros, i.e. resources being utilized only by a minority of patients, were based on results from Vuong tests and actual distribution. Zero-inflated models are nested models predicting both the occurrence of any costs (logit model) and the amount of costs (count model) [55]. Thus, these models identify reasons for higher mean costs by specifying whether (a) the proportion of patients using any service differs (logit model) or whether (b) there are any differences in the average treatment costs among those reporting at least one contact (count model). For costs associated with unemployment and disability/early retirement (binary/ordinal variables), we employed logistic regressions. Since costs related to disability/early retirement were sex-specific and thus resulted in an ordinal scale (0: no costs, 1: mean costs for males, 2: mean costs for females), we used a binary variable indicating disability/early retirement in order to conduct a logistic regression in the entire sample. Age-stratified cost analyses were illustrated by plotting the estimated costs for five consecutive age groups of similar size (18–29, 30–39, 40–49, 50–59, and 60–64). Sex-adjusted negative binomial regressions on the overall costs were run for each age group to examine the differential impact of age.

Further, descriptive measures on presenteeism were presented separately, as they could not be included in the cost estimations. Sex-stratified differences between patients with and without AD were tested by using age-adjusted zero-inflated negative binomial regressions for count variables (as above) and logistic regressions for binary variables (at least one such day).

#### Sensitivity analyses

To test for the robustness of our results, we ran one-way sensitivity analyses and varied the following assumptions: human capital approach instead of frictional cost to assess costs due to absenteeism (main calculations); only complete responses compared to complete & imputed responses (main calculations); and uniform annual inflation rates (0 % vs. 5 %) [56] compared to the sector- and year-specific rates (main calculations).

#### Additional analyses

To examine the impact of AD characteristics on the economic burden, we performed additional analyses. For both CIDI and GP diagnoses, costs were estimated separately and compared to the remaining sample without the respective diagnosis using negative binomial regressions adjusting for age and sex. Direct comparisons were not immediately feasible as GP and CIDI diagnoses did not result in two entirely distinct but in two slightly overlapping groups [7]. As indicators for AD severity, we referred to professional treatment (AD severity I; combined assessment by GP and patient), as well as number of DSM-5 AUD criteria (AD severity II; assessed by CIDI) and daily drinking levels (AD severity III; assessed by CIDI). For AD severity I, we examined excess costs associated with treatment for AD among all diagnosed patients using a binary indicator. For AD severity II and III, separate models examined the association of each continuous predictor with the respective costs among all patients. For the latter models, direct (excess) cost estimations for separate groups were not feasible as they did not imply distinct groups for which costs could be estimated for. All associations with individual costs and the respective AD characteristics were determined with age and sex adjusted negative binomial regressions for direct, indirect and overall costs.

## Results

### Sociodemographics

A detailed description of the entire APC sample can be found in previous publications (Additional file 2 of [32, 57]). For the German sample (*N* = 1,356), Table 1 presents sociodemographics by AD diagnosis. Mean patient age was 45 years and 56.9 % were female. AD diagnoses by GP or CIDI were given to 17.0 % and 6.4 % of all male and female primary care patients, respectively (total: 11.0 %). Among patients with AD diagnoses, males and patients who rated their socioeconomic status above average were overrepresented, while an average socioeconomic status was less common in this group. Further, AD cases showed higher disability levels as compared to the remaining sample. No statistically significant differences could be found for the distribution of age and unemployment.

**Table 1:** Sociodemographics and disability by AD diagnosis

	All patients	Non-AD	AD	*p* ^a^
	*N* = 1,356	*N* = 1,213	*N* = 143	
Sex (% female, CI)	56.9 (54.2–59.5)	59.8 (57.1–62.6)	33.0 (25.3–40.7)	<.001
Age (%, CI)				
18–29	19.6 (17.5–21.7)	19.1 (16.9–21.3)	23.4 (16.5–30.3)	.218
30–39	17.9 (15.9–20.0)	17.9 (15.7–20.1)	18.3 (12.0–24.6)	.908
40–49	18.7 (16.6–20.8)	19.1 (16.9–21.4)	14.8 (9.0–20.7)	.218
50–64	43.8 (41.2–46.5)	43.9 (41.0–46.7)	43.5 (35.3–51.7)	.930
Unemployment (%, CI)	12.4 (10.6–14.1)	11.8 (9.9–13.6)	17.0 (10.8–23.2)	.078
Socioeconomic status (%, CI)				
Above average	20.1 (18.0–22.3)	19.3 (17.1–21.6)	26.8 (19.5–34.1)	.037
Average	69.0 (66.5–71.5)	70.0 (67.5–72.6)	60.5 (52.5–68.6)	.021
Below average	10.9 (9.2–12.5)	10.6 (8.9–12.4)	12.7 (7.2–18.2)	.459
Disability score (WHODAS, mean, SD)	14.8 (14.7)	14.4 (14.4)	18.1 (16.2)	.004

Note

*AD* Alcohol dependence, *CI* 95 % confidence interval, *WHODAS* World Health Organization Disability Assessment Schedule 2.0

^a^difference between AD and non-AD cases was determined using Chi^2^-Tests (F-distribution) for binary variables (sex, age categories, unemployment, socioeconomic categories) and negative binomial regression for the count variable (WHODAS sum score)

### Resource use data

Resource use data were collected from the German APC study for six months before the time of interview. Descriptive summary statistics of these data by AD and sex can be found in Table 2. Among patients without AD diagnosis, three patients (0.2 %) reported treatment for alcohol problems, as compared to ten patients (7 %) in the AD group.

**Table 2:** Resource use data in six months before interview by alcohol dependence and sex

	All patients	Non-AD			AD		
	*N* = 1356	male	female	total	male	female	total
		*N* = 494	*N* = 719	*N* = 1213	*N* = 95	*N* = 48	*N* = 143
Direct costs							
Inpatient admissions ^a^ Number of nights, mean (SD)	1.2 (5.2)	1.2 (6.0)	1.1 (4.1)	1.2 (5.0)	2.5 (7.3)	0.9 (3.4)	2.0 (6.4)
Outpatient visits ^a^ At least one visit, % (CI)	9.9 (8.3–11.6)	8.7 (6.2–11.2)	9.9 (7.7–12.1)	9.4 (7.8–11.1)	15.8 (8.2–23.4)	10.1 (1.7–18.5)	13.9 (8.1–19.7)
Day case surgery visits ^a^ At least one visit, % (CI)	6.0 (4.7–7.3)	4.9 (3.0–6.8)	6.0 (4.3–7.9)	5.6 (4.3–6.9)	10.1 (3.8–16.4)	8.2 (0.5–16.0)	9.5 (4.6–14.4)
Accident & emergency visits ^a^ At least one visit, % (CI)	11.2 (9.5–12.9)	8.7 (6.2–11.2)	10.7 (8.4–13.0)	9.9 (8.2–11.6)	22.0 (13.5–30.5)	21.7 (9.8–33.6)	21.9 (15.0–28.8)
GP treatment ^a^ Number of visits, mean (SD)	6.8 (11.4)	5.8 (9.9)	6.8 (6.4)	6.4 (8.0)	10.4 (25.6)	9.6 (24.0)	10.2 (25.1)
Prescribed medication ^a^ Number of prescriptions, mean (SD)	1.8 (1.5)	1.8 (1.5)	1.9 (1.5)	1.8 (1.5)	2.1 (1.5)	1.7 (1.4)	1.9 (1.5)
Home care ^a^ At least one home visit, % (CI)	5.2 (4.0–6.4)	5.1 (3.1–7.1)	5.1 (3.5–6.8)	5.8 (3.9–6.4)	7.6 (2.2–13.1)	2.2 (0.0–6.4)	5.8 (1.9–9.8)
Alcohol treatment At least one treatment contact, % (CI)	1.0 (0.5–1.5)	0.4 (0.0–0.9)	0.1 (0.0–0.4)	0.2 (0.0–0.5)	9.6 (3.6–15.5)	2.3 (0.0–6.6)	7.2 (2.9–11.4)
Indirect costs							
Absenteeism among employed patients Number of absent days, mean (SD)	8.1 (20.4)	5.5 (15.4)	8.7 (18.4)	7.3 (17.2)	17.2 (41.1)	12.9 (26.6)	16.1 (37.9)
Unemployment % (CI)	8.2 (6.7–9.7)	7.7 (5.3–10.0)	7.8 (5.8–9.8)	7.8 (6.2–9.3)	10.5 (4.3–16.7)	13.9 (4.3–23.6)	11.7 (6.4–16.9)
Disability/early retirement % (CI)	16.5 (14.5–18.5)	17.6 (14.2–21.0)	14.7 (12.1–17.3)	15.8 (13.8–17.9)	22.2 (13.6–30.7)	21.6 (9.8–33.5)	22.0 (15.0–28.9)

Note

*AD* Alcohol dependence, *SD* Standard deviation, *CI* 95 % confidence interval, *GP* General practitioner

^a^ excluding all alcohol-specific treatments (medication, inpatient and outpatient treatment, group therapy, (GP) counselling/detoxification)

### Economic burden associated with AD

All presented costs represent the results from combining collected resource use data from the German APC study (Table 2) with German unit costs (Additional file 2). Table 3 presents the direct and indirect costs by spending sector, AD and sex. Across all patients, we have estimated that 3879.66€ of direct and indirect costs incurred per patient. Of all costs calculated for the entire sample (5405230.60€), 15 % incurred among patients with an AD diagnosis (811767.45€). The economic burden associated with AD was determined through the mean cost difference between patients with and without an AD diagnosis and equalled 1836.35€ per AD case (*p* < .001; age adjusted). In comparison to direct costs, productivity loss made up the larger share of this burden (57 % of all costs). The three largest components of the economic burden were disability/early retirement (27 %), inpatient treatment (26 %) and unemployment (20 %), which represented almost 75 % of the entire economic burden associated with AD. Alcohol treatment constituted only about 6 % of this burden.

**Table 3:** Average direct and indirect costs per patient in six months before interview in 2014-€

	All patients	Non-AD			AD			Excess costs		
	*N* = 1356	male	female	total	male	female	total	male	female	total
		*N* = 494	*N* = 719	*N* = 1213	*N* = 95	*N* = 48	*N* = 143	*N* = 589	*N* = 767	*N* = 1356
Direct costs										
Inpatient admissions ^1a^	605.53 (2446.48)	569.42 (2766.16)	543.82 (1997.69)	554.10 (2332.50)	1300.61 (3650.39)	458.99 (1634.33)	1022.95 (3165.99)	731.18 °°^−^	−84.80	468.85 °^−^
Outpatient visits ^1a^	9.78 (26.73)	8.96 (29.28)	9.08 (20.96)	9.03 (24.59)	17.03 (45.01)	13.50 (21.81)	15.86 (39.06)	8.07 °^−^	4.41	6.83 °^−^
GP treatment ^2a^	219.41 (361.28)	189.23 (321.42)	217.60 (205.77)	206.22 (326.56)	336.03 (800.45)	307.33 (749.01)	326.56 (784.31)	146.81 *^+^	89.73	120.34 *^+^
Prescribed medication ^1a^	143.45 (424.96)	157.10 (521.57)	128.18 (344.64)	139.79 (423.90)	203.94 (511.76)	110.77 (152.41)	173.21 (431.74)	46.84	−17.41	33.41
Home care ^1a^	26.69 (226.05)	17.72 (121.62)	23.98 (148.22)	21.47 (138.34)	102.80 (661.66)	0.93 (6.14)	69.19 (546.01)	85.08 *^+^	−23.05 ***^−^	47.73
Alcohol treatment ^1^	11.63 (331.60)	0.36 (8.27)	0.0 (0.0) ^d^	0.14 (5.21)	28.18 (115.97)	261.08 (1699.39)	105.01 (978.30)	27.82 °°°^−^	261.08 ^b^	104.87 ***^+^ °°°^−^
Total direct costs excluding alcohol treatment ^2^	1003.68 (2609.80)	940.67 (2931.95)	921.81 (2131.66)	929.38 (2479.83)	1960.41 (3871.48)	891.52 (1913.02)	1607.77 (3400.08)	1019.74 **^+^	−30.29	678.39 **^+^
Total direct costs including alcohol treatment ^2^	1015.31 (2628.83)	941.03 (2931.86)	921.81 (2131.66)	929.53 (2479.79)	1988.59 (3869.34)	1152.59 (2507.64)	1712.79 (3508.16)	1047.56 ***^+^	230.78	783.26 ***^+^
Indirect costs										
Absenteeism among employed patients ^1c^	680.11 (1734.23)	584.05 (1542.06)	711.72 (1742.10)	660.41 (1667.00)	932.67 (2377.10)	652.28 (1684.31)	840.17 (2180.79)	348.62 *^+^	−59.44	179.76 *^+^
Unemployment ^3^	784.20 (2626.86)	732.85 (2570.54)	750.31 (2568.01)	743.29 (2569.20)	1008.96 (2873.82)	1335.22 (3284.69)	1116.60 (3017.16)	276.12	584.91	373.31
Disability/early retirement ^3^	1402.00 (3156.06)	1445.88 (3161.93)	1281.27 (3084.52)	1347.43 (3116.68)	1824.21 (3340.81)	1888.23 (3561.13)	1845.33 (3414.18)	378.34	606.96	497.90 *^+^
Total indirect costs ^1^	2866.31 (3833.04)	2762.78 (3772.04)	2743.30 (3788.09)	2751.13 (3782.01)	3765.85 (4062.60)	3875.72 (4152.24)	3802.10 (4092.50)	1003.07 **^+^	1132.42 *^+^	1050.97 ***^+^
Total costs										
Total costs excluding alcohol treatment ^2^	3868.03 (5092.91)	3703.45 (5133.10)	3661.58 (4755.51)	3678.39 (4908.87)	5726.26 (6590.02)	4767.24 (5004.35)	5409.87 (6137.53)	2058.36 **^+^	1105.66 **^+^	1731.48 ***^+^
Total costs including alcohol treatment ^2^	3879.66 (5114.32)	3703.81 (5133.53)	3661.58 (4755.51)	3678.54 (4909.05)	5754.44 (6591.28)	5028.32 (5491.93)	5514.89 (6265.50)	2050.64 **^+^	1366.74 **^+^	1836.35 ***^+^

Note. All presented costs refer to reported and imputed costs and are presented as mean cost per patient with standard deviation in brackets

Significance of excess costs was tested with ^1^ zero-inflated negative binomial regressions or ^2^ negative binomial regressions, using alcohol dependence and age as predictors in both count (predicting values >0) and logit (predicting 0, only for zero-inflated negative binomial regressions) model. The following symbols indicate a significant AD predictor: count model * *p* < .05 ** *p* < .01 *** *p* < .001; logit model + or – behind the symbol indicates valence of respective coefficient

^3^ significance of excess costs was tested with age-adjusted logistic regressions for unemployment and disability (collapsing sex-specific costs into a single value). The same legend for p-values applies as for negative binomial models

*AD* Alcohol dependence, *GP* General practitioner

^a^ excluding all alcohol-specific treatments (medication, inpatient and outpatient treatment, group therapy, (GP) counselling/detoxification)

^b^ only one case with alcohol related treatment costs among female AD patients: *χ* ^2^-test on contingency table with one cell having 0 counts (female non-AD patients), one cell 1 count (female AD patients): *p* < .001

^c^ costs of absenteeism according to friction cost method

^d^ no costs despite resource use because one “other alcohol contact” was specified but as the type of contact was not specified, this contact was not included in the cost calculation

#### Sex- and age-stratified analyses

**Fig. 1:**
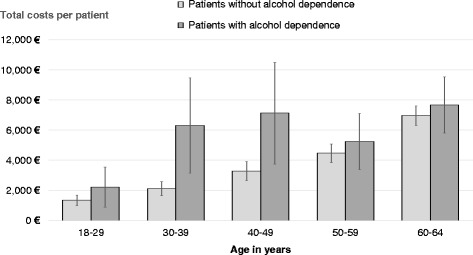
Total costs per patient by age and AD diagnosis

Sex-stratified analyses for both male and female AD patients found significant excess costs, too. Adding sex as further covariate to the age-adjusted main model (see Table 3) predicting total costs by AD diagnosis produced a non-significant effect of sex (*p* = .558; other results not shown). Fig. 1 illustrates total costs per patient by AD diagnosis for consecutive age groups, with excess costs amounting to 889.48€ (*p* = .099; 18–29 year old), 4211.53€ (*p* < .001; 30–39 year old), 3850.06€ (*p* = .006; 40–49 year old), 775.86€ (*p* = .373; 50–59 year old), and 715.26€ (*p* = .269; 60–64 year old).

#### Direct costs

We found higher direct costs due to health care utilization among AD cases irrespective of including or excluding alcohol treatment. Overall, 18 % of all direct costs incurred among patients with an AD diagnosis. Inpatient treatment – not including specialized alcohol-related hospitalization – produced the highest share of mean direct excess costs (468.85€), followed by GP treatment (120.34€) and alcohol-specific treatment (104.87€).

According to the results from the zero-inflated model, excess costs in inpatient admissions were likely to be due to a greater proportion of male AD cases reporting any inpatient admission (logit model), while differences in the costs of admissions were not found (count model). For home care related costs (excess costs per male AD case: 85.08€), the results were the other way round, with male AD patients being just as likely to report any home care (logit model), but if they received any home care, their costs were higher (count model) than those of other male patients.

Results of direct mean cost differences among female patients with or without an AD diagnosis were quite mixed and not significant in most sectors, except for reception of home care services. Only one female AD case reported receiving some kind of home care, thus resulting in significant lower costs compared to other female patients (−23.05€ per female AD case). Alcohol treatment was found to make up the largest share of excess costs among female AD cases (261.08€ per case), but this was mainly due to a single case reporting 40 inpatient nights which resulted in a large mean and standard deviation. Overall, the excess direct costs associated with AD equalled 230.78€ per female patient but were not significantly different from zero.

#### Indirect costs

**Fig. 2:**
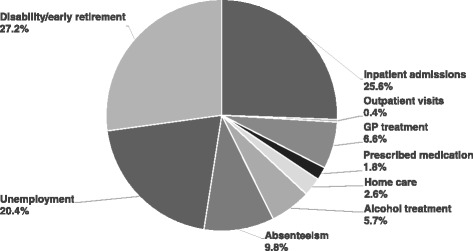
Share of excess costs of alcohol dependence by sector

Indirect costs due to absenteeism, unemployment and disability or early retirement were found to be concentrated among AD cases, which were responsible for 14 % of all indirect costs. On average, these costs were 1,050.97€ higher among AD cases as compared to other patients. This finding was also reproduced in sex-stratified analyses but not for all components of indirect costs. In total, indirect costs account for more than half of all observed excess costs, which is also demonstrated by Fig. 2.

We found that presenteeism was also more prevalent among AD cases (see Table 4). Male patients with an AD diagnosis reported more days with reduced work capacity due to any health condition (WHODAS 2.0) or due to mental distress (K10) as compared to non-AD cases. Further, for both male and female patients, alcohol-associated work impairment was present. While about 4 % of all cases with a gainful occupation perceived their productivity to be affected by their drinking, this rate was significantly higher among AD cases (22 %).

**Table 4:** Descriptive measures of presenteeism among gainfully occupied patients

	All patients	Non-AD			AD		
	*N* = 792	male	female	total	male	female	total
		*N* = 317	*N* = 403	*N* = 720	*N* = 52	*N* = 20	*N* = 72
Number of days of reduced work in past 30 days due to any health condition (WHODAS 2.0 H3), mean (SD)	3.0 (6.1)	2.3 (5.1)	3.3 (6.2)	2.9 (5.8)	4.0 (8.2) *^+^	3.6 (7.5)	3.9 (8.0) *^+^
Number of days with at most half of usual work capacity in past 30 days due to mental distress (K10 Q6), mean (SD)	2.0 (4.4)	1.4 (3.2)	2.1 (4.1)	1.7 (3.8)	3.2 (7.2)**^+^	6.7 (8.7)	4.1 (7.7) ***^+^
Impaired work productivity due to drinking in past six months							
At least one day, proportion (95 % CI)	3.8 (2.5–5.2)	4.2 (2.0–6.5)	0.3 (0.0–0.8)	2.0 (1.0–3.0)	24.1 (12.2–35.9) ***^+^	16.0 (0.0–32.7) **^+^	21.9 (12.1–31.7) ***^+^
Number of days, mean (SD)	0.3 (3.1)	0.1 (0.8)	0.0 (0.1)	0.1 (0.5)	2.9 (10.8) **^+^	0.4 (1.2) °°^−^	2.3 (9.4) **^+^ °^−^

Note

Significance between patients with and without AD was tested with zero-inflated negative binomial regressions for all count variables, using alcohol dependence and age as predictor in both count (predicting values >0) and logit (predicting 0) model. The following symbols indicate a significant AD predictor: count model * *p* < .05 ** *p* < .01 *** *p* < .001; logit model ° *p* < .05 °° *p* < .01 + or – behind the symbol indicates valence of respective coefficient

For the binary variable indicating at least one day with impaired productivity due to drinking, an age-adjusted logistic regression was conducted. The same legend for p-values also applies to negative binomial models

*AD* Alcohol dependence, *WHODAS* World Health Organization Disability Assessment Schedule 2.0, *SD* Standard deviation, *K10* Kessler Psychological Distress Scale, *95* % *CI* 95 % confidence interval

### Sensitivity analysis

A one-way sensitivity analysis was conducted, varying assumptions on costs due to absenteeism, imputation of missing values, and annual inflation rates (see Table 5). Results suggest that extent and significance of mean excess costs among AD cases remained significant under all observed conditions.

**Table 5:** One-way sensitivity analyses: variation of assumptions on costs of productivity, imputed values, and inflation rates

	Human capital approach			Only unimputed costs			Annual inflation rate 0 %			Annual inflation rate 5 %		
	Non-AD	AD	Total	Non-AD	AD	Total	Non-AD	AD	Total	Non-AD	AD	Total
	*N* = 1,213	*N* = 143	*N* = 1,356	*N* = 1,213	*N* = 143	*N* = 1,356	*N* = 1,213	*N* = 143	*N* = 1,356	*N* = 1,213	*N* = 143	*N* = 1,356
Direct costs												
Total direct costs excluding alcohol treatment ^1^	/	/	/	867.28 (2466.25)	1559.27 (3405.17) **^+^	943.07 (2599.48)	800.91 (1950.23)	1371.41 (2715.12) **^+^	863.40 (2059.54)	1171.80 (3393.09)	2035.01 (4551.12) **^+^	1266.34 (3554.46)
Total direct costs including alcohol treatment ^1^	/	/	/	867.42 (2466.21)	1659.84 (3511.19) ***^+^	954.21 (2618.22)	801.06 (1950.18)	1476.12 (2857.76) ***^+^	875.00 (2084.39)	1171.96 (3393.04)	2141.91 (4624.28) ***^+^	1278.19 (3567.83)
Indirect costs												
Absenteeism ^2^	941.91 (3004.53)	1893.80 (6667.85) **^+^	1046.24 (3626.33)	/	/	/	654.52 (1652.13)	832.67 (2161.33) *^+^	674.04 (1718.76)	687.24 (1734.74)	874.31 (2269.40) *^+^	707.74 (1804.70)
Total indirect costs ^2^	3032.64 (4400.97)	4855.73 (7102.00) ***^+^	3232.44 (4820.05)	/	/	/	2738.60 (3766.75)	3784.64 (4073.89) ***^+^	2853.25 (3817.31)	2808.17 (3852.92)	3881.61 (4179.12) ***^+^	2925.82 (3906.19)
Total costs												
Total costs excluding alcohol treatment ^1^	3959.68 (5504.37)	6463.50 (9368.64) ***^+^	4233.91 (6119.86)	3616.29 (4889.88)	5361.37 (6139.02) ***^+^	3807.41 (5077.34)	3537.41 (4582.74)	5156.05 (5629.39) ***^+^	3714.69 (4740.96)	3977.80 (5583.99)	5916.62 (7106.93) ***^+^	4190.15 (5809.62)
Total costs including alcohol treatment ^1^	3959.83 (5504.52)	6568.52 (9442.82) ***^+^	4245.54 (6137.20)	3616.43 (4890.06)	5461.94 (6268.48) ***^+^	3818.56 (5098.91)	3537.56 (4582.94)	5260.77 (5772.92) ***^+^	3726.29 (4764.30)	3977.96 (5584.17)	6023.52 (7212.45) ***^+^	4202.00 (5828.11)

Note. All presented costs refer to mean cost per patient with standard deviation in brackets

Imputations mainly affect the prescribed medication section, in addition to inpatient treatment, home care, and alcohol treatment

Significance of excess costs was tested with ^1^ negative binomial regressions or ^2^ zero-inflated negative binomial regressions using alcohol dependence and age as predictors in both count (predicting values >0) and logit (predicting 0, only for zero-inflated negative binomial regressions) model. The following symbols indicate a significant AD predictor: count model * *p* < .05 ** *p* < .01 *** *p* < .001; logit model ° *p* < .05 °° *p* < .01 °°° *p* < .001. + or – behind the symbol indicates valence of respective coefficient

*AD* Alcohol dependence. / = same value as in Table 1, i.e. not affected by human capital approach or by imputations

Using the human capital approach yielded expectedly higher indirect costs via absenteeism, with total costs being 8.6 % greater following this approach as compared to the frictional cost approach. Excluding all imputed values from cost estimations produced small changes in direct costs (−6.4 %) and total costs (−1.6 %). Compared to sector- and year-specific inflation rates (main calculations), estimating the economic burden assuming 0 % or 5 % inflation led to a 4.1 % decrease and a 5.5 % increase, respectively.

### Additional analysis

Results from additional analyses examining the impact of the diagnostic approach (GP vs. CIDI) and of different AD severity indicators are presented in Table 6. A significant economic burden was observed regardless of the diagnostic approach, with significantly higher indirect costs associated with both diagnoses. Direct costs were found to be significantly higher among GP diagnosed patients but not among CIDI diagnosed patients.

**Table 6:** Association of AD characteristics and individual costs

	Type of AD diagnosis ^a^		AD severity I ^b^	AD severity II ^c^	AD severity III ^c^
	Excess costs: GP AD	Excess costs: CIDI AD	Excess costs: AD treatment	IRR: DSM-5 AUD criteria	IRR: Daily drinking levels
	*N* = 1,356	*N* = 1,356	*N* = 143	*N* = 1,356	*N* = 1,356
All direct costs (95 % CI)	1132.20 (198.64–2066.09)**	322.07 (−303.93–948.08)	−17.89 (−1336.70–1300.92)	1.09 (0.99–1.19)	0.99 (0.95–1.03)
All indirect costs (95 % CI)	1947.09 (950.67–2943.52)**	355.04 (−510.20–1220.28)*	1148.41 (−688.51–2985.33)	1.12 (1.04–1.20)**	1.01 (0.98–1.04)
All costs (95 % CI)	3081.46 (1526.96–4635.96)***	679.20 (−560.11–1918.52)**	1130.52 (−1435.05–3696.09)	1.11 (1.04–1.19)**	1.00 (0.98–1.03)

Note

*AD* Alcohol dependence, *GP AD* Alcohol dependence diagnosis by general practitioner (*N* = 70), *CIDI AD* Alcohol dependence diagnosis by the Composite International Diagnostic Interview (*N* = 92), *IRR* Incidence risk ratio, *DSM*-*5 AUD criteria* Number of alcohol use disorder criteria as defined in the Diagnostic and Statistical Manual of Mental Disorders, 5th edition, *95* % *CI* 95 % confidence interval

For testing impact of AD characteristics on different cost variables, age and sex adjusted negative binomial regressions were conducted. Legend of significance: * *p* < .05 ** *p* < .01 *** *p* < .001

^a^ Excess costs for GP AD and CIDI AD diagnoses are presented in comparison to patients without the respective diagnosis. Results based on regression using binary predictor (no diagnosis/diagnosis) among all patients

^b^ Excess costs between treated and untreated AD cases are presented. Results based on regression using binary predictor (untreated/treated) among all AD cases

^c^The incidence risk ratios from negative binomial regressions are presented for DSM-5 AUD criteria and drinking levels. Results based on regression using continuous predictor (severity II: number of DSM-5 AUD criteria/ severity III: daily drinking levels in standard drinks per day) among all patients

Results of the association of various AD severity indicators and incurred costs were mixed. For AD severity I, costs were not significantly different between treated and untreated AD cases. Similarly, no significant association between daily drinking levels (AD severity III) and individual costs was observed for any of the examined cost domains. However, the only significant association was established for the number of AUD criteria (AD severity II) and indirect as well as total costs: The more AUD criteria were met, the higher costs incurred.

## Discussion

### Summary

This study sought to estimate the economic burden associated with AD using a bottom-up approach with a sample of 1356 German primary care patients. Our estimations considered direct and indirect costs separately and additional analyses examined the impact of several AD characteristics on respective costs.

In the sample studied, the economic burden of AD was determined using excess costs which amounted to about 1867€ per case. In other words, the direct and indirect costs among AD patients were about 50 % higher compared to primary care patients without AD. Age- and sex-stratified analyses showed that these costs were concentrated among males and patients aged 30 to 50. Excess costs were largely attributable to indirect costs (57 %) in addition to costs related to inpatient treatment (25 %). Costs reported for different kinds of alcohol treatment made up a relatively small share, amounting to 6 % of the excess costs. Sensitivity analysis suggests that the estimates are robust under various assumptions.

Additional analyses indicate that increased costs were independent of the approach to diagnose AD, as overall costs were significantly higher among both GP and CIDI diagnosed primary care patients, as compared to non-AD cases. Furthermore, our results suggest that costs may increase with AD severity but this finding was limited to the number of AUD criteria and could not be reproduced with treatment of AD and drinking levels.

### Comparison with top-down estimations

In comparison with previous top-down estimations, the costs per patient and the breakup of direct and indirect costs were very similar [11, 12, 21], which supports the validity of studies using top-down methods. A more detailed look at the results reveals that indirect costs were consistently found to account for the majority of all costs, in this sample (57 %), in a German study on alcohol-associated costs (65 %) [12], and in a systematic review on AD-associated economic burden in high-income countries (72 %) [11]. However, top-down estimations usually included costs due to premature mortality in their estimations, e.g. being accountable for 45 % of all alcohol-associated costs in the above-mentioned German study. If we adjusted indirect cost estimations in the other studies to the same definition as used in our study by excluding premature mortality, the share of indirect costs would be reduced markedly to only 20 % in the German study and to 52 % across various high-income countries. Thus, the reported indirect costs in our sample were comparably high, especially compared to the German study. This discrepancy may be explained by differences between this sample and general population studies, with primary care patients being older and having more mental and physical health issues, which is related to unemployment and claims of disability benefits or early retirement pensions [58, 59]. Further, to grant any sick leave in Germany, employers commonly ask their employees to provide certification of illness from a medical professional, linking absenteeism closely to GP visits.

### Implications

Our results suggest that the overwhelming majority of the economic burden was not due to the direct treatment of AD but more than 80 % was attributable to paid absence from work, disability or early retirement, and inpatient treatment of comorbid health problems. Given the low treatment rates for AD (17.2 % in this sample, see [57]) and a non-significant impact of AD treatment on any cost dimension (see Additional analyses), it is not surprising that the share of excess costs attributable to alcohol treatment is relatively small. Low treatment rates imply lower costs for alcohol-specific interventions but also result in high comorbidity and continuing impairment. In fact, previous studies have suggested to increase treatment coverage in order to bring about significant reductions in morbidity and mortality [60, 61], which we would expect to lead to a reduced economic burden.

Apart from costs for alcohol treatment, inpatient treatment and GP visits made up most of the excess direct costs. While alcohol is a component cause of over 200 health conditions [23, 62], AD is associated with a wide range of physical comorbidities in inpatient settings [8], and the present sample of AD cases was found to be comorbid with liver diseases and severe mental distress [57]. Thus, highly frequent health care resource utilization and associated costs among AD cases are not surprising and most likely not only attributable directly to alcohol problems, but to a significant extent to comorbidities associated with or caused by this condition. Therefore, our findings highlight and reiterate the necessity of preventing heavy drinking over time in order to reduce respective detrimental health consequences.

Half of the excess costs were due to increased rates of unemployment, early retirement, and disability. Further, absenteeism was also identified as important contributor to excess costs. While presenteeism was not part of cost calculations in this study, it was very common among AD cases. Some guidelines suggested the inclusion of presenteeism in the calculation of indirect costs [63] while others suggested not to do so [38]. In fact, many published studies on cost-of-illness of alcohol neglected this factor e.g. [2, 12, 15, 17] and only very few studies included presenteeism in their estimations e.g. [64]. Our work suggest that presenteeism is associated with AD and should therefore be included in future studies.

A major implication of this study concerns the observation that impaired productivity represents the largest share of the economic burden associated with AD, confirming previous top-down estimations from Germany [12, 65] and elsewhere (for a review, see [66]). Public health efforts should aim at reducing this burden while acknowledging the bidirectional association of unemployment and heavy drinking [67, 68] and strategies to do so in primary health care settings have been proposed [69].

Sex-stratified cost estimations in our study suggest that excess costs mainly incurred among male AD cases in our study. This is not only due to males representing about two thirds of all AD cases, but rather to generally larger cost differences in most sectors between both sexes: Total direct costs were significantly increased in male but not in female AD cases, and excess costs were almost doubled among male compared to female AD cases. While we corroborate that female AD patients are less likely to receive treatment for their alcohol problems than male AD patients are [70], the greater picture is rather surprising. As the small number of females in this sample implies large confidence intervals and susceptibility to outliers, these results should be carefully interpreted as trends. However, the results point out that sex-stratified cost estimations are warranted and future research should follow up these preliminary observations. Distinct sex-specific patterns of mental [71] and somatic comorbidities [23] should be considered as potential explanations for differential cost discrepancies found in this study.

Furthermore, our results indicate that most of the excess costs incurred among individuals aged 30 to 49, while no substantial cost differences was observed in younger or older cohorts. This effect is interesting and could likely be linked to mortality, which has been shown to be constantly high among young and middle aged individuals with alcohol use disorders, as opposed to the general population, for which mortality increases steadily with age [4]. High excess costs in combination with high mortality in these age groups support the need of initiating treatment earlier – before somatic comorbidities emerge and become life-threatening [28].

Lastly, results from additional analyses show that the economic burden is not only associated with DSM-IV AD diagnoses but also with GP AD diagnoses, which can be viewed as a validation for their diagnostic quality. In addition, DSM-5 AUD severity was also found to be associated with increased economic burden. Associations with direct costs were only found for GP AD diagnoses and future research should examine why associations with other AD characteristics, including treatment and drinking levels, remained insignificant. In this study, small sample sizes cannot be excluded as potential cause.

### Limitations

For a variety of reasons, the estimates in this study are not directly comparable to a general population cost-of-illness study. The primary reason is that this study referred to a representative sample of primary care patients, which differs considerably in its distribution of sex and health measures from the general population. Further, the reported estimations do not consider intangible costs and costs associated with premature disability and presenteeism – measures that should be considered when estimating the economic burden of AD to the whole society. In addition, further wider social costs are evident as a result of alcohol related crime, both to the criminal justice system and also victim costs and further injury-related productivity costs through absenteeism. Although these go beyond the remit of the current study, crime costs form a substantial proportion of alcohol related societal costs [72–74]. Taken together, the figures of this study are likely underestimates and should not be generalized to the general population. Further, stratified analyses of age, sex, and AD characteristics should be interpreted cautiously as subgrouping results in smaller samples, which are more susceptible to outliers and thereby to erroneous inferences.

With regard to valuating productivity losses, we used the friction cost approach for absenteeism and considered transfer payments (benefits, taxation, insurances) for unemployment, disability and early retirement. This was a conservative decision avoiding the potential overestimation of the true cost of productivity losses as if using the human capital approach, and was believed to better represent the real costs of productivity losses than with the human capital approach, which shows the potential consequences of disease for an economy in a state of full employment equilibrium. The friction approach is also believed to prevent adverse equity implications [51, 56].

## Conclusions

Despite the mentioned limitations, the present study corroborates the importance of the economic burden associated with AD as determined by previous general population top-down estimates by using a bottom-up approach: Total costs were 50 % higher among patients with AD compared to patients without this diagnosis (excess costs: 1836€ per case), with excess costs mainly attributable to reduced productivity. We found high concentrations of AD-associated costs among males and patients aged 30 to 50. Furthermore, DSM-5 AUD severity was also significantly linked to the economic burden. While we generally confirm previous estimations of AD-associated economic burden, we have also identified several discrepancies. Future cost-of-illness studies using a bottom-up approach in a general population sample may be able to examine possible reasons for these gaps, and they should consider presenteeism in their estimations.

## Additional files

Additional file 1: Service Use Questionnaire adapted from the UK Alcohol Treatment Trials. (PDF 678 kb) Additional file 2: Web Table 1: Description of unit costs in 2014-€. Web Figure 1: Estimating costs related to prescribed medication. (PDF 388 kb)

## Abbreviations

AD: Alcohol dependence
APC study: ‘Alcohol dependence in primary and specialist care in Europe’ study
DSM-IV: Diagnostic and statistical manual of mental disorders, fourth edition
GPs: General practitioners
DSM-5: Diagnostic and Statistical Manual of Mental Disorders, fifth edition
AUD: Alcohol use disorder
CIDI: Composite international diagnostic interview
WHODAS: World Health Organization Disability Assessment Schedule
K10: Kessler psychological distress scale
UKATT: United Kingdom Alcohol Treatment Trial
