# Supplementary material for: Economic burden associated with alcohol dependence in a German primary care sample: a bottom-up study
Source: BMC Public Health. 2016 Aug 31;16(1):906. doi: 10.1186/s12889-016-3578-8 (PMC5006576; doi:10.1186/s12889-016-3578-8)
Supplement: Additional file 1: — Service Use Questionnaire adapted from the UK Alcohol Treatment Trials. (PDF 678 kb) [file 12889_2016_3578_MOESM1_ESM.pdf]

Service use questionnaire

Adapted from the UK Alcohol Treatment Trials for the APC study

HOSPITAL ADMISSIONS DURING PERIOD

Was the patient admitted to hospital **during the last 6 months**?

Yes ☐1 **No** ☐2

Accident & Emergency (A&E) EPISODES

Was the patient admitted to a **hospital A & E Department during the last 6 months?**

Yes ☐1 **No** ☐2

***If Yes**, please supply the following information for each admission.*

	**Admission 1**	**Admission 2**	**Admission 3**
**1. What happened to the patient following treatment?**	☐ Patient sent home same day<br>☐ Overnight stay in A & E<br>☐ Patient admitted as inpatient	☐ Patient sent home same day<br>☐ Overnight stay in A & E<br>☐ Patient admitted as inpatient	☐ Patient sent home same day<br>☐ Overnight stay in A & E<br>☐ Patient admitted as inpatient

DAY CASES

Was the patient admitted to hospital **during the last 6 months for day case surgery**?

Yes ☐1 **No** ☐2

***If Yes**, please supply the following information for each admission.*

	Admission 1	Admission 2	Admission 3
**1. Department? (refer to annexe)**	☐☐	☐☐	☐☐

INPATIENT CARE

Was the patient admitted to hospital **during the last 6 months as an inpatient**?

Yes ☐1 **No** ☐2

***If Yes**, please supply the following information for each admission.*

	Admission 1	Admission 2	Admission 3
**1. Department? (refer to annexe)**<br><br>**Was this for alcohol treatment?**	☐☐<br><br>**Yes** ☐<br>**No** ☐	☐☐<br><br>**Yes** ☐<br>**No** ☐	☐☐<br><br>**Yes** ☐<br>**No** ☐
**2. How many nights did the patient spend in hospital?**	☐☐	☐☐	☐☐

OUTPATIENT CARE

Was the patient admitted to hospital **during the last 6 months as an outpatient**?

Yes ☐1 No ☐2

***If Yes**, please supply the following information for each admission.*

	Episode 1	Episode 2	Episode 3
**1. Department? (refer to annexe)**	☐☐	☐☐	☐☐

GENERAL PRACTICE SERVICE USE

1. Has the patient used any of the following services during the last 6 months (excluding treatment for a drinking problem) ?

Yes ☐1 No ☐2

If ***Yes***, please estimate the total number of contacts for each service during the last 6 months

(i) G. P. - Surgery Visit ☐☐

(ii) G. P. - Home Visit ☐☐

(iii) Practice Nurse (at GP surgery) ☐☐

2. Have you received any prescriptions over the last 6 months?

Yes ☐1 No ☐2

Please list below any medication which you have received over the last six months, and how many prescriptions you have received for each.

(i) ☐☐

(ii) ☐☐

(iii) ☐☐

(iv) ☐☐

(v) ☐☐

OTHER SERVICE RECEIPT

1. Has the patient used any of the following services during the last 6 months? **Yes ☐1 No ☐2**

		No of contacts
**(i)**	**Community Psychiatric Nurse (at home)**	
**(ii)**	**Social Worker (at home)**	
**(iii)**	**Occupational Therapist (at home)**	
**(iv)**	**District Nurse (at home)**	
**(v)**	**Support Worker (at home)**	
**(vi)**	**Other (please specify)**	

ALCOHOL SERVICES

1. **Has the patient sought help for drinking problems in the last 6 months?**

Yes ☐1 **No ☐2**

Please indicate any contacts with the following types of services regarding your drinking problem over the last *6 months*

		Number of appointments kept	Type of care
**(i)**	this agency		Individual care ☐<br>Group care ☐
**(ii)**	another alcohol agency		Individual care ☐<br>Group care ☐
**(iii)**	residential rehabilitation		Individual care ☐<br>Group care ☐
**(iv)**	hospital		Individual care ☐<br>Group care ☐
**(v)**	counsellor/nurse in a GP surgery		Individual care ☐<br>Group care ☐
**(vi)**	a self help group		Individual care ☐<br>Group care ☐
**(vii)**	other (please detail)		Individual care ☐<br>Group care ☐

2. Has your treatment with an alcohol service resulted in an overnight stay over the last *6 months*?

Yes ☐1 No ☐2

If Yes, please indicate the number of nights spent in treatment.

(i) residential rehabilitation ☐☐

(ii) hospital inpatient ☐☐

(iii) other ☐☐

3. Have you received help from your GP regarding a drinking problem over and above those contacts listed earlier over the last *6 months*?

Yes ☐1 No ☐2

If yes, please indicate the number of contacts for the following types of care.

(i) counselling ☐☐

(ii) detoxification ☐☐

(iii) other ☐☐

EMPLOYMENT

1. How many hours do you work on average per week? ☐☐

2. Have you had to take any days off from work over the last 6 months? **Yes** ☐1 **No** ☐2

If Yes, please estimate the number of days absence from work during this period ☐☐

3. Have you had an accident or caused any damage at work in the last 6 months as a result of your drinking?

Yes ☐1 **No** ☐2

4. Do you think your performance at work been affected as a result of drinking over the past 6 months ?

Yes ☐1 **No** ☐2

On how many days over the last 6 months has your productivity at work been affected ? ☐☐

Slightly ☐1
Moderately ☐2
Considerably ☐3
Extremely ☐4

ANNEXE

CODES: HOSPITAL DEPARTMENTS

Medical	Code
• Paediatrics	01
• Geriatrics	02
• Cardiology	03
• Dermatology	04
• Infectious disease	05
• Medical oncology	06
• Neurology	07
• Rheumatology	08
• Gastroenterology	09
• Haemotology	10
• Clinical Immunology & Allergy	11
• Thoracic Medicine	12
• Genito-urinary Medicine	13
• Nephrology	14
• Rehabilitation Medicine	15
• Other Medicine	16
**Surgical**	
• General Surgery	17
• Urology	18
• Orthopaedics	19
• E.N.T.	20
• Opthamology	21
• Gynaecology	22
• Dental specialties	23
• Plastic Surgery	24
• Cardiothoracic	25
• Paediatric	26
**Maternity**	
• Obstetrics	27
• General Practice	28
**Psychiatric**	
• Mental Handicap	29
• Mental Illness	30
• Psychotherapy	31

- **Old age psychotherapy** **32**

Other

- **General Practice** **33**
- **Radiotherapy** **34**
- **Pathological Specialties and Radiology** **35**
- **Anaesthetics** **36**
