# Supplementary material for: Economic burden associated with alcohol dependence in a German primary care sample: a bottom-up study
Source: BMC Public Health. 2016 Aug 31;16(1):906. doi: 10.1186/s12889-016-3578-8 (PMC5006576; doi:10.1186/s12889-016-3578-8)

Article title: Economic burden associated with alcohol dependence in a German primary care sample: A bottom-up study

Web Table 1.

Description of unit costs in 2014-€.

	Original year of unit costs	Unit cost (2014-€)	Description of unit costs	Source of unit costs
**Resource use component - direct costs**				
Hospital attendance				
One inpatient admission	2000/2003	334.03 - 515.62	Range of costs per night, depending on department of admission	Krauth et al. (2005)
One outpatient visit	1999/2003	19.76 - 393.93	Range of costs per admission, depending on department admitted to	Krauth et al. (2005)
One day case surgery visit	1999/2003	19.76 - 393.93	Treated same as outpatient	Krauth et al. (2005)
One accident & emergency visit	1999/2001	515.62 / 47.90	Treat as inpatient if night spent in department / otherwise treat as outpatient	Krauth et al. (2005)
General practice				
Any practice visit	2012	31.21 / 36.17	Up to age 59 / at least 60 years old, costed via EBM adjusted for share of private health insurance[1]	Kassenärztliche Bundesvereinigung (2012)<br>Krauth et al. (2005)
Home visit	2012	52.48 / 57.44	Up to age 59 / at least 60 years old, costed via EBM adjusted for share of private health insurance[1]	Kassenärztliche Bundesvereinigung (2012)<br>Krauth et al. (2005)
Home care:<br>Any contact at home with…				
Occupational therapist	2015	45.25	Mean of costs for different services + charge for home visit, adjusted for share of statutory and private health insurance	KV Berlin (2015)<br>Vdek (2015)<br>Krauth et al. (2005)
Alternative practitioner	2002	40.62	Based on common charge for home visit	Fachverband Deutscher Heilpraktiker (2002)
Physiotherapist	2014	38.02	Based on Krauth et al (2015), inflated to 2014 € + charge for home visit from vdek (2015)	Krauth et al (2005)<br>vdek (2015)
Community psychiatric nurse, social worker, other health professionals	2014	42.36	Based on average of the three services above	See above
Paid homemaker	2014	20.01	Based on average gross income of 'other labour forces'	GENESIS-Online (2015)
Prescribed medication				
Cost per day * intake length	2014	0.03 - 7328.21<br>Median : 34.84	Range of costs of one medication determined in the sample; costed via N3 pharmacy prices	Rote Liste ® (2015)

Alcohol treatment (not involving overnight stays)				
GP contact at practice	2012	31.21 / 36.17	As above	Krauth et al. (2005) Kassenärztliche Bundesvereinigung (2012)
Outpatient contact	2014	177.45	e.g. in specialized alcohol agencies or hospitals, treat as semi-residential contacts	DRG Research Group (2015)
Counselling contact	1999	60.05	e.g. in specialized alcohol agencies, treat as psychotherapy	Krauth et al. (2005)
Group therapy	2014	48.70	e.g. self-help group, costed as opportunity costs of one leisure hour for each patient and therapist, unit costs determined via gross mean wage	GENESIS-Online (2015)
Alcohol treatment (involving overnight stay(s))				
First night	2014	549.59	Logarithmic growth of costs for overnight stay; maximum costs for 180 days: 39,211.66 €	DRG Research Group (2015)
**Resource use component - indirect costs**				
Costs of one day absent from work – human capital	2013	243.46 / 205.32	For male / female; mean costs of one working hour multiplied with mean daily working hours	Statistisches Bundesamt (2014)
Costs of one day absent from work – frictional cost	2013	194.77 / 164.26	80% of the above within friction period (49 days)	Krauth et al. (2005)
Six months unemployed	2013	9,576.92	Based on paid benefits and reduced tax and insurance fee revenue	Institut für Arbeitsmarkt und Berufsforschung (2014)
Six months disabled/in early retirement	2014	8,228.38 / 8,738.38	For male / female; mean costs of reduced tax and insurance fee revenue and paid benefits for people with reduced working capacity for east German federal states	Institut für Arbeitsmarkt und Berufsforschung (2014) Deutsche Rentenversicherung (2015)

Note.
EBM: = standard evaluation citeria (‘Einheitlicher Bewertungsmaßstab‘)
[1] adjustment for share of private health insurance was necessary if costs were provided from statutory health insurance

Web Figure 1

Estimating costs related to prescribed medication

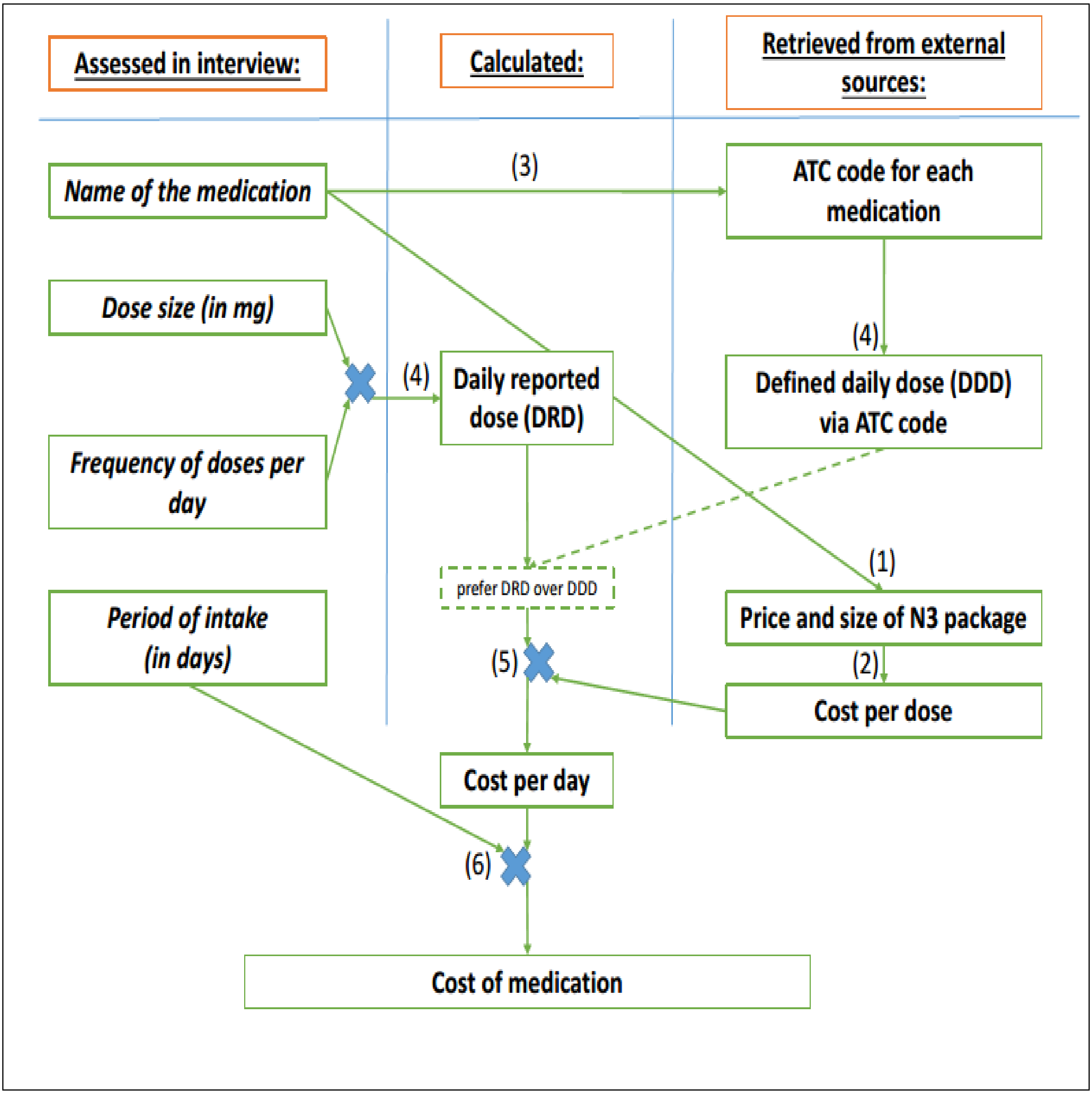

Supplement: Additional file 2: — Web Table 1: Description of unit costs in 2014-€. Web Figure 1: Estimating costs related to prescribed medication. (PDF 388 kb) [file 12889_2016_3578_MOESM2_ESM.pdf]
